# Motor Development of Premature Infants Born between 32 and 34 Weeks

**DOI:** 10.1155/2010/462048

**Published:** 2010-09-07

**Authors:** S. A. Prins, J. S. von Lindern, S. van Dijk, F. G. A. Versteegh

**Affiliations:** ^1^Department of Pediatrics, Groene Hart Ziekenhuis, P.O. Box 1098, 2800 BB Gouda, The Netherlands; ^2^Department of Neonatology, Leiden University Medical Center, P.O. Box 9600, 2300 RC Leiden, The Netherlands; ^3^Department of Physical Therapy, Groene Hart Ziekenhuis, P.O. Box 1098, 2800 BB Gouda, The Netherlands

## Abstract

Little is known about motor development in late preterm born infants. Our objective was to determine long-term outcome of motor skills of infants born between 32 and 34 weeks. All infants were assessed at corrected ages of 3 and 9 months, using the Alberta Infant Motor Scale. At corrected ages of 4 years, the Movement Assessment Battery for Children was done. Seventy infants were seen at 4 years of age (median of 3 assessments per infant). Abnormal assessment at 3 or 9 months of age resulted in normal outcome in almost 80% at 4 years. On the other hand, a normal outcome in the first year of life resulted in an abnormal outcome at 4 years in 10% of the infants. Our results suggest that long-term followup of these late preterm born infants is necessary, as the assessments in the first year do not predict the long-term outcome.

## 1. Introduction

Premature born infants are at increased risk for morbidity, longer hospital stay, and death as opposed to term born infants [1, 2]. Much of the recent neonatal outcome literature focuses on infants with a very low birth weight (<1500 gram) or infants born very prematurely (<32 weeks gestation) [3–5]. Several studies have mentioned lower cognitive scores, increased risk for Attention Deficit Hyperactivity Disorder, and delayed motor development in premature infants with very low birth weight [3–8]. However, the group of infants born between 32 and 37 weeks of gestation is larger. In the Netherlands approximately 30% of all infants are born at home, almost all at term. Of the infants born in hospitals, approximately 7% to 11% are born between 32 and 37 weeks of gestation [7]. In the literature mainly short-term outcome has been described in this age group [9]. It is known that infants born between 32 and 34 weeks have a four times greater risk of needing mechanical ventilation, ten times greater risk of developing a pneumothorax, and a 15 times greater risk of developing a sepsis as opposed to term born infants [1, 2, 10, 11]. There are only a few studies on long-term motor developmental outcome, that is, several years after hospital discharge, in infants born between 32 and 34 weeks [2, 12, 13]. 

Our hypothesis was that preterm infants born between 32 and 34 weeks are at risk for delayed motor development.

## 2. Patients and Methods

From January 1999 till January 2005, we included all 126 infants born between 32 0/7 and 34 0/7 weeks admitted to our neonatology ward, a medium and high care unit. During their stay, follow-up of motor development was offered by the attending physician and physical therapist as part of the post clinical developmental care program. We studied their motor development at the corrected ages of 3, 6, and 9 months and 4 years. Written parental consent was obtained to describe the results in a manuscript. As follow-up of the psychomotor development is part of our standard care for premature born infants it was not necessary to ask for approval from the institutional medical ethical committee. 

### 2.1. Intake and Assessments

Four experienced and trained physical therapists participated in this study. No infant had physical therapy a priori. During the initial hospital stay one of the physical therapist took a full history and performed a first exam. After discharge, the Alberta Infant Motor Scale (AIMS) was performed by one of the physical therapists at the corrected age of 3, 6 and 9 months. At the corrected age of 4 years the children were assessed using the Movement Assessment Battery for Children (M-ABC). The infants were assessed at the hospital in a separate room during 45 minutes, of which 20 minutes was pure observation time. The infants were randomly assessed by one of the physical therapists using the standardized AIMS and M-ABC forms. All physical therapists recorded the data in the same Access file. 

#### 2.1.1. Alberta Infants Motor Scale (AIMS)

The Alberta Infants Motor Scale (AIMS) is a reliable norm-referenced observational tool that has been validated for use from term to 18 months of age in infants both born premature and full term [14]. AIMS measures qualitative aspects of movements without touching the infant and is sensitive to changes in an infants motor behavior [14]. It also identifies infants who are delayed in their gross motor development. After observation of the infant, a raw AIMS score is based on the number of gross motor positions and movements that the infant has shown while being prone, supine, sitting, and standing. This raw score is translated to a percentile ranking that can be compared with normative age-matched samples of infants. Since there are no Dutch norm values yet [15], we used the cutoff point of the tenth percentile, as recommended by Darrah et al. [14]. Below the tenth percentile the motor development was classified as abnormal.

### 2.2. Movement Assessment Battery for Children (M-ABC)

At the corrected age of 4 years, motor development was assessed using Movement Assessment Battery for Children [16]. The M-ABC is a norm-referenced test that consists of 32 tasks divided into four age bands. For this study, we used band 1 (4–6 years). The tasks are divided into three sections: manual dexterity, ball skills, and static and dynamic balance. A child's score on each test is converted to a standardized impairment score, based on centile bands for the child's age group. An infant has a normal score when the total impairment score (TIS) is at or above the 16th percentile. Infants with a TIS <16 percentile were considered to be abnormal.

### 2.3. Statistical Analysis

In the final analysis we included patients for whom 2 or more assessments, of which one was the M-ABC, were performed. The data were analyzed using SPSS for Windows (version 17.0; SPSS, Chicago, IL). To determine the correlation between AIMS and M-ABC, we used the Spearman's rho correlation test. Statistical differences were considered significant if *P* < .05.

## 3. Results

From January 1999 until January 2005, 126 inborn infants, born after a gestational age between 32 0/7 and 34 0/7 weeks were admitted to the neonatology ward of the Groene Hart Ziekenhuis, Gouda, the Netherlands and assessed by our physical therapists. All parents gave their informed consent for this study. The patient characteristics are described in Table 1. None of the children had serious abnormalities on cranial ultra sound (10 had flaring grade 1 [17] in the first week only, and two infants had an enlarged ventricle but still within normal values according to Levene [18]. None of the infants required mechanical ventilation or inotropic support. Ninety percent of the patients of the study group were infants of Caucasian parents. As far as recorded, 4% of the families had a lower socio-economic status (SES). In the majority of 70 infants (51 infants), the existence of chorioamnionitis was not tested. Only 2/19 mothers were diagnosed with a chorioamnionitis. The numbers concerning SES and chorioamnionitis are too small for statistical analysis. Fourty-two infants (60%) received breast milk for any period of time. Of the infants who did not receive breast milk 7.4% had abnormal scores at 4 years of age, as opposed to the infants with breast milk where 21.4% had abnormal motor development at 4 years of age.

**Figure 1:**
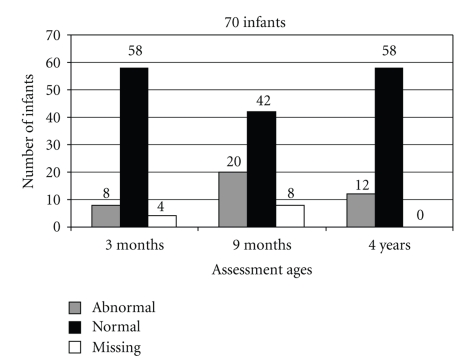
Motor developmental scores of patients at different corrected ages.

Of 70/126 infants (55%), the M-ABC at 4 years of age was done next to at least one other assessment in the first year of life (median of 3 assessments). A reason for the high number of drop-outs is not known. The subgroup of infants with assessments at 4 years of age and at least one in the first year was further analyzed. Due to a very limited number of assessments at 6 months of age, these results are not described. The outcome of all assessments is shown in Figure 1. At 3 months of age 8/66 infants assessed (12%) showed an abnormal motor development, only one of these infants still had an abnormal development at four years of age. At the corrected age of 9 months, 20/62 infants scored abnormal, of which 20% (4 infants) still scored abnormal at 4 years of age. The percentage of children with a normal motor development was 68% and 83%, respectively at 9 months and 4 years of corrected age. There was no statistical correlation between the motor development at 3 months or 9 months and 4 years (*P* = .26 and *P* = .21, resp.).

Six infants had an Apgar score below 7 after 5 minutes (6%). At 3 months, one of these infants scored abnormal but normal at four years of age. At the corrected age of 4 years another infant scored abnormal at the M-ABC. Only one infant was small for gestational age and showed a normal motor development. Girls tended to perform better than the boys. Due to the small numbers, no significance testing was performed.

## 4. Discussion

An abnormal assessment at 3 or 9 months of age evolved to normal motor developmental outcome in 80% at 4 years of age. On the other hand, 10% of the children who scored normal in the first year showed a delayed/abnormal motor development at the age of four years. Our results suggest that long-term follow-up of these late preterm born infants is necessary, as the assessments in the first year do not predict the long-term outcome. Our results show that almost 20% of the infants born late preterm has an abnormal assessment at the age of 4, which is comparable with the outcome of ELBW infants [19].

Variables as SES, presence of chorioamnionitis, breast milk, and ethnicity have been described to be of influence on motor development [20–22]. In our study there seems to be no effect of gestational age, birth weight, Apgar score, SES, or sex on motor developmental outcome, but the numbers were too small for statistical analysis. In our study pathological exam of the placenta was not routinely done, leading to too small numbers for analysis. In our study group, on the contrary to other studies, breast milk was not protective for abnormal motor development. It is possible that we did not find the same results as other studies because of the small study group. Gestational age range in this study was also very narrow which could also explain that no effect was seen for this item. Only one child was small for gestational age. 

In the literature, some authors have described the use of the AIMS in late preterm infants. Restiffe and Gherpell, described the use of the AIMS in 43 infants, ranging from 26 to 36 5/7 weeks [13]. All infants were assessed at chronological ages. They concluded that all premature infants should be assessed at corrected ages to prevent children from being wrongly diagnosed with developmental delay. No specific results of the late preterm infants were mentioned separately. Fleuren et al. assessed 100 children using the AIMS. They found that Dutch children in the age of 0–12 months scored significantly lower on the AIMS than the Canadian reference group of 1990–1992 [15]. Also, they stated that the AIMS is the most sensitive in the first year of life. Another Dutch group, Van Haastert et al., assessed 800 preterm infants with the AIMS and found evidence of a specific way of early motor developmental in the first 18 months of life of these infants. They suggested that standard gross motor developmental scales should be adjusted for preterm infants [8]. 

As shown by the above-mentioned authors it is difficult to compare preterm infants to term infants. Furthermore, our results show that infants that have been assessed in the first year of life and scored abnormal can evolve to normal motor development at four years of age. On the other hand, a normal score the first year can lead to abnormal motor development later on. It is possible, however, that the sample size of our study is too small to rule out chance in this respect. It is possible that the percent with abnormal motor development at each age is within the range expected by chance with this sample size. As there are no data described for this age group, we can not compare this. 

Our study has some limitations. There was a relatively high rate of loss to follow up in the total group of 126 infants. Of the group with an assessment at four years, several infants were not assessed at all study moments in the first year. Possibly, these infants had motor developmental difficulties and may have been seen by physical therapists nearby their home, whose assessments were not available for this study. Another possibility is that the infants were developing well and the parents did not see the necessity of a motor assessment. Second limitation is not blinding the investigators to the gestational age, which may have led to a bias. However the physical therapists used two objective assessment tools. 

Our study results show that assessments in the first year of life in late preterm infants do not predict their motor developmental outcome at 4 years of age. However, we do suggest assessments at 3, 9, months and at 4 years of age. Assessments in the first year seem warranted to establish motor developmental delay, in order to provide for the most optimal support in the motor development of this potentially vulnerable group of infants. Possibly assessment at a later age (for instance at 7 to 9 years) is warranted to evaluate later outcome in fine motor skills.

## Figures and Tables

**Table 1:** Patient characteristics.

	Inborn infants	Study group	*P*
Patients (*n*)	126	70	—
Male/female, *n* (% male)	84/42 (67)	51/19 (73)	—
Gestational age (weeks), median (IQR)	33 (32 2/7–33 5/7)	33 1/7 (32 3/7–33 6/7)	.18
Birth weight (gram) median (IQR)	1918 (1666–2143)	2000 (1750–2170)	.08
Apgarscore 5′ median (IQR)	9 (8–10)	9 (8–10)	.33
Apgarscore 5′ < 7, *n* (%)	8 (6.3%)	6 (8.5%)	.53
